# Informationen zur medizinischen Vorgeschichte in der Notaufnahme: Einfluss auf Therapie- und Diagnostikentscheidungen

**DOI:** 10.1007/s00063-020-00661-8

**Published:** 2020-02-10

**Authors:** M. Lorsbach, A. Gillessen, K. Revering, C. Juhra

**Affiliations:** 1 Stabsstelle für Telemedizin, Universitätsklinikum Münster, Hüfferstraße 73–79, 48149 Münster, Deutschland; 2 Klinik für Innere Medizin, Herz-Jesu-Krankenhaus Hiltrup GmbH, Münster-Hiltrup, Deutschland; 3 Zentrale Notaufnahme, Herz-Jesu-Krankenhaus Hiltrup GmbH, Münster-Hiltrup, Deutschland

**Keywords:** Elektronische Patientenakte, Notfalldatensatz, Telematik, Anamnese, Notfallmedizin, Electronic health record, Emergency care data set, Telematics, Medical history taking, Emergency medicine

## Abstract

**Hintergrund:**

Die Einführung einer elektronischen Patientenakte (ePA) bzw. eines Notfalldatensatzes (NFD) ist ebenso wie die Reform der Notfallversorgung in Deutschland derzeit immer wieder Teil politischer Diskussionen. Derzeit existieren in Deutschland keine Daten zum Nutzen einer solchen ePA bzw. NFD für die Notaufnahmen. Ziel dieser Studie war es herauszufinden, ob mitgebrachte Vorbefunde Einfluss auf Diagnostik- und Therapieentscheidungen in der Notaufnahme haben.

**Methodik:**

Zur Beantwortung der Frage wurde eine deskriptive Beobachtungsstudie in einer interdisziplinären Notaufnahme durchgeführt mit einer Studienpopulation von *n* = 96.

**Ergebnisse:**

Hinsichtlich vorhandener Vorbefunde konnten bei 55 Patienten (59 %) weder ein Arztbrief noch eine Medikamentenliste gefunden werden. Jedoch konnten bei 48 % der Patienten, die über die Notaufnahme stationär aufgenommen wurden, Ergänzungen der Anamnese nachgewiesen werden.

Bei 8 (9 %) Patienten zeigte sich, dass Therapie- und/oder Diagnostikentscheidungen hätten diskutiert bzw. geändert werden müssen, falls die ergänzten anamnestischen Informationen in der Notaufnahme vorgelegen hätten. Die Dauer der Anamnese zeigte sich ebenfalls verlängert bei fehlenden Vorbefunden seitens des Patienten (Mittelwert: 10–15 min; Standardabweichung: ±<5 min) im Gegensatz zu den Patienten mit Vorbefunden (Mittelwert: 5–10 min; Standardabweichung: ±<5 min).

**Diskussion:**

Mithilfe von ePA und NFD könnten Therapie- und Diagnostikentscheidungen sicherer gestellt werden. Beim Fehlen von Vorbefunden ist die Anamnesedauer in Notaufnahmen deutlich verlängert, was sich durch die Einführung einer ePA bzw. eines NFD reduzieren ließe.

## Abstract (en)

**Background:**

The introduction of an electronic health record (EHR) or an emergency care data set (ECDS), as well as reforms in emergency medical care, is currently part of political debate in Germany. Currently, no data are available of how emergency departments could benefit from an ePA or NFD in Germany. The aim of this study was to determine if a patient’s medical history has an influence on diagnostic and therapeutic decisions in the emergency department.

**Methodology:**

To answer this question, a descriptive observational study was conducted in an interdisciplinary emergency department with a study population of *n* = 96.

**Results:**

For 55 patients (59%) neither a doctor’s letter nor a drug list was found. However, in 48% of the patients who were admitted to the hospital via the emergency department, additions to the anamnesis record could be identified. Eight (9%) patients showed that therapy and/or diagnostic decisions should have been discussed or changed if the supplemented anamnestic information had been available in the emergency room. In addition, the study revealed that the duration of the anamnesis was prolonged in case of missing medical history (mean: 10–15 min, standard deviation: ±<5 min). In contrast to the patients with a medical history (mean: 5–10 min, standard deviation: ±<5 min).

**Conclusion:**

Based on the data stored in EHR and ECDS, therapy and diagnostic decisions could be made more reliably. In the absence of a medical history, the time required for medical history taking in emergency departments is significantly longer, which could be reduced by introducing EHR or ECDS.

Die Zuständigkeit für die Versorgung von medizinischen Notfällen obliegt in Deutschland sowohl den niedergelassenen Ärzten, dem Rettungs- und Notarztdienst als auch den Notaufnahmen der Kliniken [1]. Alle diese Institutionen benötigen Informationen bezüglich des Patienten, um eine bestmögliche Versorgung zu gewährleisten. Studien zeigen, dass von einer jährlichen Steigerung der Anzahl an Notfallpatienten in den Notaufnahmen von über 9 % auszugehen ist [5]. Hierbei ist, vor dem Hintergrund der demographischen Entwicklung, ein Anstieg besonders der älteren Notfallpatienten zu erwarten. Eine Arbeitsgruppe der Deutschen Gesellschaft für Interdisziplinäre Notfall- und Akutmedizin (DGINA) kommt in einer Studie auf einen Anteil von über 30 % der über 70-jährigen Notfallpatienten [4]. Besonders bei diesen Patienten ist von einer komplexen medizinischen Vorgeschichte und Hausmedikation auszugehen. Vor diesem Hintergrund wurde 2003 das E‑Health-Gesetz verabschiedet, um die Qualität der Versorgung auf einem hohen Niveau zu halten und um Arbeitsabläufe effizienter zu gestalten [7]. Besonders im Bereich der präklinischen Notfallversorgung soll daher ein Notfalldatensatz eingeführt werden, um eine zielgerichtete präklinische Diagnostik und Therapie zu vereinfachen. Zusätzlich plant die Bundesregierung eine Reform der Notfallversorgung. Inhalte dieser Reform soll eine gemeinsame Notfallleitstelle, erreichbar unter 112 oder 116.117, sein sowie die Errichtung von Notfallzentren in Kliniken mit ärztlichem Notdienst, gestaltet durch die kassenärztliche Vereinigung. Daneben bilden Notaufnahmen weiterhin die Versorgung für vital bedrohte Patienten ab. Ebenso soll der Rettungsdienst künftig ein eigenständiger medizinischer Leistungsbereich werden [2].

Vorbefunde können in einer Notfallsituation sehr nützlich sein. Eine elektronische Patientenakte (ePA) bzw. ein Notfalldatensatz (NFD) auf der Gesundheitskarte könnten hier zukünftig dem Personal der Notaufnahmen helfen, diese wichtigen Informationen schnell und mit minimalem Aufwand zur Verfügung zu haben. Die Einführung einer ePA bzw. des NFD ist jedoch mit einem erheblichen technischen und personellen Aufwand verbunden, sodass die Frage erlaubt sein muss, welchen potenziellen Nutzen diese Instrumente für die Notaufnahme bieten.

Ziel dieser Studie war es herauszufinden, ob mitgebrachte Informationen, ähnlich wie die Inhalte des Notfalldatensatzes, Einfluss auf Diagnostik- und Therapieentscheidungen in der Notaufnahme haben. Hierzu sollten folgende Fragen beantwortet werden. Als primäre Fragestellungen dieser Studie wurden definiert:

- Mit welchen Informationen zur medizinischen Vorgeschichte kommt der Patient?
- Wurden anamnestische Informationen im Rahmen des Erstkontakts in der Notaufnahme oder im Fall einer weiteren stationären Behandlung initial oder im Verlauf ergänzt?
- Hätten die ergänzten anamnestischen Informationen Einfluss auf Therapie- und Diagnostikentscheidungen gehabt?

Als sekundäre Fragestellungen wurden definiert:

- Mussten Informationen aktiv (beispielweise durch Telefonate mit Hausärzten etc.) in der Notaufnahme ermittelt werden?
- Wie viel Zeit nahmen die Anamnese und die ggf. erforderliche Informationsbeschaffung in Anspruch?
- Wurde die Verweildauer in der Notaufnahme durch mitgebrachte Vorbefunde beeinflusst?

## Methodik

Zur Beantwortung der Fragestellung: „Welchen Einfluss hat die Anamnese des Notfallpatienten in der Notaufnahme auf die Diagnostik- und Therapieentscheidung?“, wurde eine prospektive deskriptive Beobachtungstudie durchgeführt.

## Studiendesign

**Figure 1:**
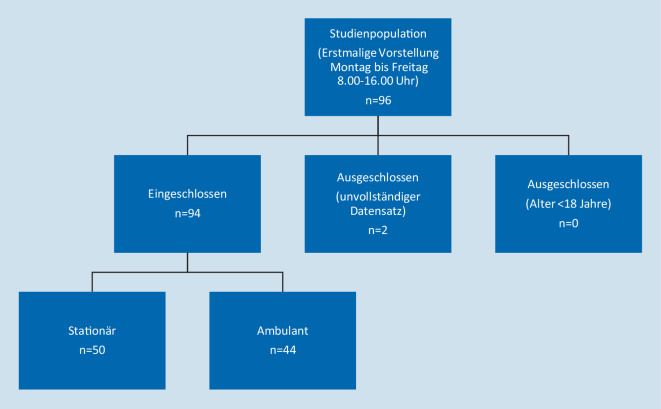

Als Studienpopulation (Abb. 1) wurden *n* = 96 Patienten untersucht. Es wurde eine Notaufnahme ausgewählt, die zentral geführt wird und die Hauptfachabteilungen der Allgemein- und Unfallchirurgie, der inneren Medizin mit den Schwerpunkten Gastroenterologie, Nephrologie und Kardiologie, der Neurologie mit zertifizierter Stroke-Unit, Urologie und Gynäkologie mit Geburtshilfe aufwies. Einschlusskriterien waren alle Patienten, die notfallmäßig und erstmalig in der zentralen Notaufnahme von montags bis freitags in der Zeit zwischen 8.00–16.00 Uhr (August bis September 2018) vorstellig wurden. Die Datenerhebung erfolgte über einen zusammenhängenden Zeitraum von 4 Wochen. Aufgrund von unvollständig ausgefüllten Checklisten in Teil 1 wurden 2 Patienten aus der Studie ausgeschlossen. Als Ausschlusskriterien waren ein Alter unter 18 Jahren und das Vorliegen von Informationen in Altakten über den Patienten definiert.

## Erhebungsassessment

**Figure 2:**
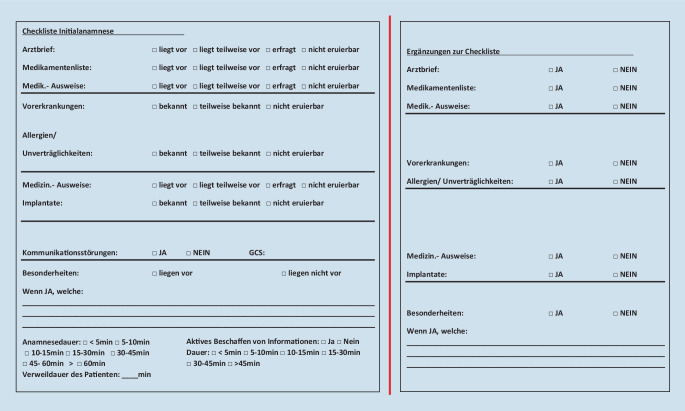

Die Checkliste zur Erhebung der Daten gliederte sich in 3 Anteile. Der 1. Teil (Abb. 2) bestand in der Erhebung der Informationen zum Zeitpunkt der initialen Anamnese in der Notaufnahme. Bei den Items Arztbrief, Medikamentenliste, Medikamentenausweise und medizinische Ausweise konnte zwischen *liegt vor, liegt teilweise vor, erfragt *und* nicht eruierbar* ausgewählt werden. Bei diesen Items handelte es sich um Informationen, die in gedruckter Form zum Zeitpunkt der Anamnese bereits durch den Patienten vorgelegt wurden. Bei der Auswahl *liegt vor* wurde ein vollständig gedrucktes Schriftstück seitens des Patienten vorgelegt. Die Auswahl *liegt teilweise vor* wurde erhoben, wenn Auszüge aus einem Arztbrief oder ein vom Patienten selbst erstellter Medikamentenplan vorlag. Ebenso wurden Überweisungsscheine mit einer kurzen Epikrise der akuten Symptomatik als *liegt teilweise vor* gewertet. *Erfragt* wurde hingegen ausgewählt, wenn Inhalte zu Arztbriefen, Medikamentenlisten, Medikamentenausweisen und medizinischen Ausweisen durch gezieltes Nachfragen ermittelt wurden oder der Patient angab, dass ein Item existierte oder nicht, aber nicht vorgelegt werden konnte. Als *nicht eruierbar* wurde das Item bezeichnet, wenn keinerlei Informationen seitens des Patienten zu erheben waren. Des Weiteren wurde der Bekanntheitsgrad für den Patienten zu seinen Vorerkrankungen, Allergien, Unverträglichkeiten und Implantaten ermittelt. Hier wurde *bekannt, teilweise bekannt* und *nicht eruierbar* zur Auswahl angeboten. Die Frage nach Kommunikationsstörungen wurde mit *Ja* oder *Nein* beantwortet. Hierbei wurde eine Kommunikationsstörung definiert als die Unmöglichkeit der adäquaten Anamnese des Patienten aufgrund von sprachlichen Barrieren oder Bewusstseinseinschränkungen. Bei der Beurteilung von Kommunikationsstörungen wurde auch die Glasgow Coma Scale des Notfallpatienten bei Eintreffen berücksichtigt. Hierbei wurde das Patientenbewusstsein in leichte, mittlere und schwere Störungen eingeteilt [8]. Bei der Erfassung des Patientenbewusstseins konnten Besonderheiten, falls vorhanden, in einem Freitext festgehalten werden.

Ein aktives Beschaffen von Informationen während des Aufenthalts des Patienten in der Notaufnahme konnte mit „Ja“ oder „Nein“ beantwortet werden. Zusätzlich erfasst wurde die Dauer, die das Beschaffen von Informationen benötigte. Gemessen wurde dabei die Zeit, die aktiv aufgebracht werden musste, um an zusätzliche Informationen von extern zu gelangen. Zudem wurden die Zeiten für die Anamnesedauer und die Verweildauer des Patienten in der Notaufnahme ermittelt.

Im 2. Teil (Abb. 2) der Checkliste wurde die Anamnese auf etwaige Ergänzungen hin überprüft. Diese Ergänzungen stammten entweder aus der aktiven Ermittlung von Informationen während des Aufenthalts in der Notaufnahme oder aus Informationsbeschaffungen im Verlauf des stationären Aufenthalts. Die Überprüfung fand bei den stationär aufgenommenen Patienten ca. 5 Tage nach Aufnahme mithilfe der Patientenakte statt. Hierbei konnten die Ergänzungen zum Arztbrief, Medikamentenliste, Medikamentenausweise, Vorerkrankungen, Allergien und Unverträglichkeiten, medizinische Ausweise und Implantate mit *Ja *oder *Nein* ermittelt werden. Zudem gab es ein Freitextfeld für Besonderheiten, die bei den Ergänzungen der Anamnese auftraten.

**Figure 3:**
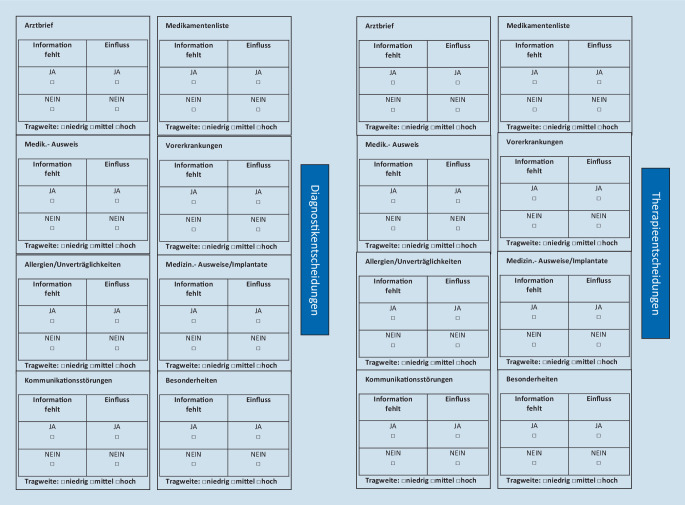

Im 3. Teil der Checkliste (Abb. 3) wurde überprüft, ob die ergänzten Informationen Einfluss auf die Diagnostik- und Therapieentscheidungen in der Notaufnahmesituation gehabt haben. Falls ein Einfluss ermittelt wurde, wurde diesem eine Tragweite von *niedrig, mittel* oder *hoch* zugeordnet. Hierbei bedeutet eine *niedrige* Tragweite, dass sich keine relevanten Diagnostik- oder Therapieänderungen ergeben hätten. Eine Tragweite von *mittlerer *Qualität bedeutet, dass weitergehende Maßnahmen mit einem Facharzt diskutiert worden wären und es im Einzelfall zu Diagnostik- oder Therapieänderungen gekommen wäre. Wurde die Tragweite mit *hoch* eingeschätzt, dann hätte es eine Änderung hinsichtlich der Diagnostik- und Therapieentscheidungen in der Notaufnahme gegeben.

## Untersuchungsablauf

Nach Anmeldung des Patienten bei der Patientenannahme wurde ermittelt, ob der Patient zum 1. Mal zu einer Behandlung in die Notaufnahme kam. Dies wurde mithilfe des Krankenhausinformationssystems EDV-gestützt durchgeführt.

Erfüllte ein Notfallpatient die o. g. Einschlusskriterien, wurden die ärztliche Anamnese hinsichtlich der zu erhebenden Parameter beobachtet und unter Zuhilfenahme des oben beschriebenen Erhebungsassessments die notwendigen Daten erfasst. Die Verweildauer der Patienten in der Notaufnahme wurde durch das Krankenhausinformationssystem EDV-gestützt erhoben. Die Überprüfung der Anamnese auf Ergänzungen wurde bei stationären Behandlungen im weiteren Behandlungsverlauf ca. 5 Tage nach stationärer Aufnahme anhand der elektronischen als auch der papiergeführten Patientenakte durchgeführt.

Nach Kontrolle der Checkliste auf Vollständigkeit in Teil 1 und Teil 2 wurde der Einfluss der gewonnenen Informationen auf die Patientenbehandlung mit dem zuständigen Arzt der jeweiligen Abteilung oder mit dem ärztlichen Leiter der Notaufnahme besprochen. Bei der Einschätzung der Einflussnahme war stets der Facharztstatus der jeweiligen Abteilung gegeben.

## Ergebnisse

Insgesamt erfüllten 94 Patienten die Einschlusskriterien und konnten in die Studie aufgenommen werden. Bei 50 Patienten ergab sich im weiteren Verlauf ein stationärer Behandlungsbedarf und 44 Patienten konnten ambulant behandelt werden.

### Mit welchen Informationen zur medizinischen Vorgeschichte kommt der Patient?

Bei 19 Patienten (20 %) lagen alle zu ermittelten Vorbefunde in Form eines Arztbriefs und einer Medikamentenliste vor. 2 hatten einen Arztbrief (2 %), 10 eine Medikamentenliste (11 %). Ein inkompletter Arztbrief lag bei 11 Patienten (12 %) und bei 63 Patienten (67 %) nicht vor. Eine Medikamentenliste lag bei 3 Patienten (3 %) teilweise und bei 62 Patienten (66 %) nicht vor. Somit konnten 55 Patienten (59 %) weder einen Arztbrief noch eine Medikamentenliste zum Zeitpunkt der Anamnese in der Notaufnahme vorlegen (vgl. Tab. 1).

**Table 1:** 

	Liegt vor	Liegt teilweise vor	Erfragt	Nicht eruierbar	Gesamt
Arztbrief	21 (22 %)	11 (12 %)	58 (62 %)	4 (4 %)	94 (100 %)
Medikamentenliste	29 (31 %)	3 (3 %)	59 (63 %)	3 (3 %)	94 (100 %)
Medikamentenausweise	1 (1 %)	0 (0 %)	86 (91 %)	7 (7 %)	94 (100 %)
Medizinische Ausweise	1 (1 %)	0 (0 %)	86 (91 %)	7 (7 %)	94 (100 %)
	Bekannt	Teilweise bekannt	Nicht eruierbar		
Vorerkrankungen	76 (81 %)	15 (16 %)	3 (3 %)	–	94 (100 %)
Allergien/Unverträglichkeiten	85 (90 %)	6 (6 %)	3 (3 %)	–	94 (100 %)
Implantate	87 (93 %)	1 (1 %)	6 (6 %)	–	94 (100 %)
		Ja	Nein		
Kommunikationsstörungen	–	9 (10 %)	85 (90 %)	–	94 (100 %)
Besonderheiten	–	0	94 (100 %)	–	94 (100 %)

### Wurden anamnestische Informationen im Rahmen des Erstkontakts in der Notaufnahme oder im Fall einer weiteren stationären Behandlung initial oder im Verlauf ergänzt?

**Figure 4:**
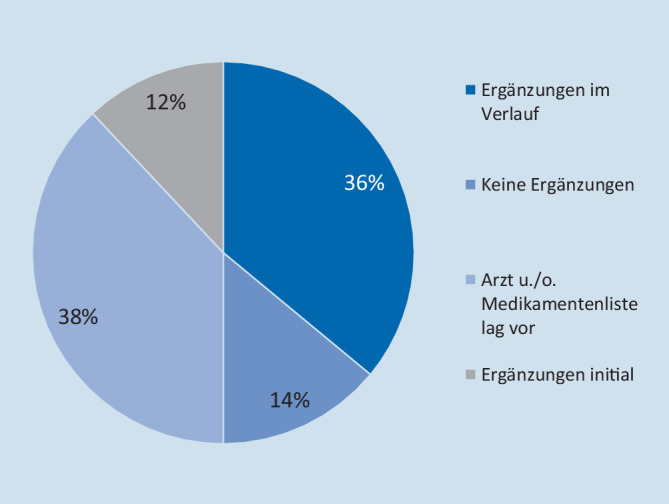

Von den Patienten mit stationärer Behandlung kamen 31 ohne Vorbefunde (62 %) im Sinne eines Arztbriefs oder einer Medikamentenliste. 19 Patienten (38 %) brachten Vorbefunde mit. Noch in der Notaufnahmesituation wurden durch Bemühungen des Aufnahmearzts mit entsprechendem Zeitaufwand bei 6 Patienten (12 %) Vorbefunde ermittelt. Während des weiteren stationären Aufenthalts wurden bei weiteren 18 Patienten (36 %) Vorbefunde beschafft, sodass am Ende des stationären Aufenthalts bei 43 Patienten (86 %) Vorbefunde vorlagen. In 7 der Fälle mit stationärem Behandlungsbedarf (14 %) fanden keine Ergänzungen zur Anamnese statt (vgl. Abb. 4). Bei den in der Notaufnahme ambulant behandelten Patienten wurde eine Information im Rahmen des Erstkontakts ergänzt.

### Hätten die ergänzten anamnestischen Informationen Einfluss auf Therapie- und Diagnostikentscheidungen gehabt?

Die Gruppe mit ergänzten Informationen zählte *n* = 31 und somit 33 % der 94 in die Studie eingeschlossenen Patienten. Durch die nachträglich hinzugewonnenen Informationen wurde in 6 von den 31 Fällen (19 %) eine Therapieänderung und in 7 Fällen (23 %) eine Diagnostikänderung als indiziert angesehen. Insgesamt konnten 11 Informationen mit Einfluss auf Diagnostikentscheidungen bei den 7 oben genannten Fällen ermittelt werden. Bei den Therapieentscheidungen konnten 10 Informationen in 6 oben genannten Fällen mit Einfluss ermittelt werden. Die Tragweiten der einzelnen ergänzten Informationen auf Diagnostikentscheidungen verteilten sich gleichmäßig auf *niedrig* und *mittel* für Patienten mit stationärem Behandlungsbedarf. Bei einem ambulant behandelten Patienten wurde die Tragweite der ergänzten Information mit *hoch* angegeben. Bei den 9 ergänzten Informationen mit Einfluss auf Therapieentscheidungen wurde bei den Patienten mit stationärem Behandlungsbedarf eine *niedrige* Tragweite 4‑mal und eine *mittlere* Tragweite 5‑mal angegeben (vgl. Tab. 2 und 3). Im Ambulanten Bereich wurde eine ergänzte Information mit Einfluss auf Therapieentscheidungen mit der Tragweite *hoch* angegeben.

**Table 2:** 

Einfluss stationär JA	9	
Einfluss ambulant JA	1	
Tragweite stationär:	*Niedrig*	4 (45 %)
	*Mittel*	5 (55 %)
	*Hoch*	0
Tragweite ambulant	*Niedrig*	0
	*Mittel*	0
	*Hoch*	1 (100 %)

**Table 3:** 

Einfluss stationär JA	10	
Einfluss ambulant JA	1	
Tragweite stationär:	*Niedrig*	5 (50 %)
	*Mittel*	5 (50 %)
	*Hoch*	0
Tragweite ambulant	*Niedrig*	0
	*Mittel*	0
	*Hoch*	1 (100 %)

### Mussten Informationen aktiv (beispielweise durch Telefonate mit Hausärzten etc.) in der Notaufnahme ermittelt werden?

**Figure 5:**
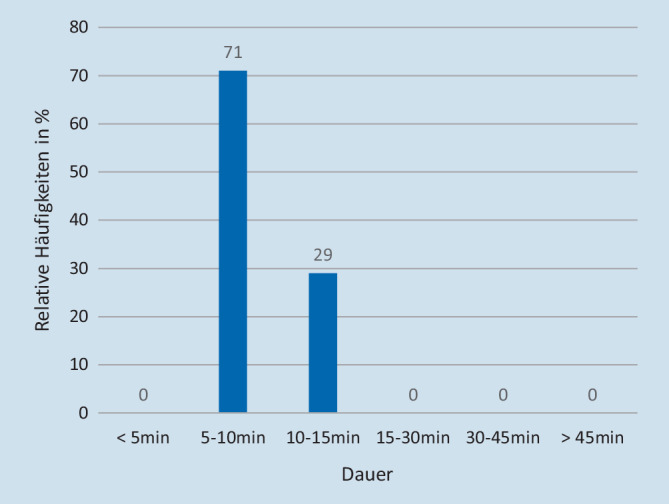

**Figure 6:**
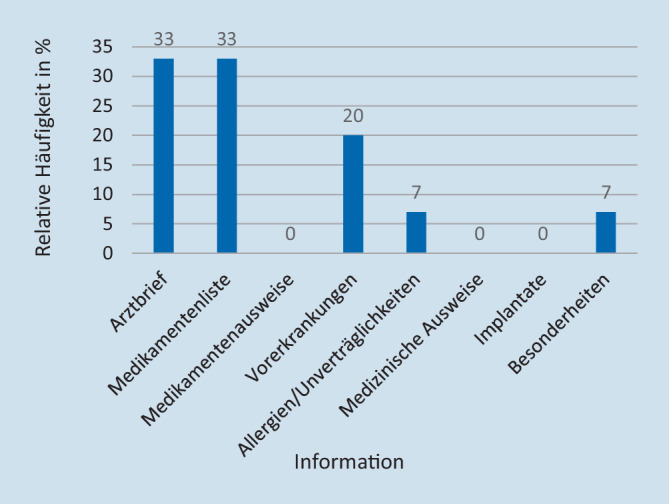

Zu einer aktiven Ermittlung von Informationen durch beispielweise Telefonate mit dem Hausarzt kam es bereits in der Notaufnahmesituation bei insgesamt 7 (7 %) Patienten. Der Zeitbedarf für die Ermittlung von Vorbefunden durch den aufnehmenden Arzt lag zwischen 5 (Minimum) und 15 min (Maximum; Mittelwert [MW]: 5–10 min; vgl. Abb. 5). Die aktiv ermittelten Informationen waren am häufigsten ein früherer Arztbrief und eine Medikamentenliste. Zusätzlich wurden auch Vorerkrankungen, Allergien und Unverträglichkeiten und eine Information unter der Rubrik „Besonderheiten“ ermittelt (vgl. Abb. 6).

### Wie viel Zeit nahmen die Anamnese und die ggf. erforderliche Informationsbeschaffung in Anspruch?

**Figure 7:**
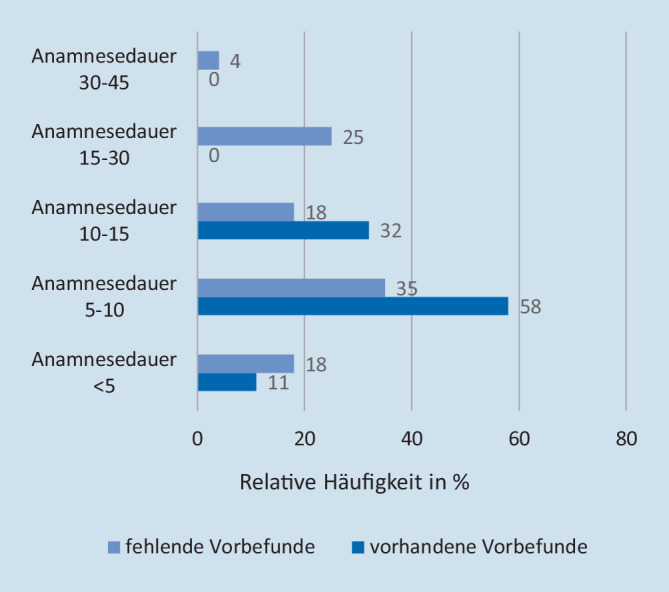

Die mittlere Anamnesedauer des Gesamtkollektivs betrug 10–15 min (Standardabweichung [SD]: ±<5 min). In der Gruppe der Patienten, die sowohl einen Arztbrief als auch ein Medikamentenliste vorlegen konnten, befand sich die mittlere Anamnesedauer am häufigsten im Zeitfenster von 5–10 min (SD: ±<5 min). Keine Anamnese bei dieser Patientengruppe benötigte länger als 15 min. Hingegen findet sich in der Gruppe der Patienten ohne mitgebrachte Vorbefunde die Anamnesedauer am häufigsten im Zeitfenster von 10–15 min (SD: ±<5 min; vgl. Abb. 7). Bezüglich der Analyse der Anamnesedauer war es ohne Unterschied, ob die Patienten stationär oder ambulant behandelt wurden.

### Wurde die Verweildauer in der Notaufnahme durch mitgebrachte Vorbefunde beeinflusst?

Die mittlere Verweildauer betrug insgesamt 155 min (SD: ±84 min). Wurden Vorbefunde im Sinne eines Artbriefs oder einer Medikamentenliste vorgelegt, betrug die mittlere Verweildauer 176 min (SD: ±94 min). In den Fällen von fehlenden Vorbefunden wurde eine mittlere Verweildauer von 149 min (SD: ±68 min) ermittelt.

## Diskussion

Aufgrund der Untersuchungsergebnisse lässt sich die These aufstellen, dass bei Fehlen eines Arztbriefs und/oder einer Medikamentenliste der zeitliche Aufwand zur Anamneseerhebung in Notaufnahmen deutlich verlängert ist und es somit zu einem vermeidbaren Zeitverlust des ärztlichen Personals und infolge dessen zu einer ineffizienteren Patientenbehandlung kommen könnte. Eine kanadische Studie zeigte, dass von 1002 Notfallpatienten in der Notaufnahme bei 404 Patienten Informationslücken gefunden werden konnten [10]. Hierbei waren bei 57 % aller Patienten die Krankengeschichte des Patienten mit Informationslücken am meisten vertreten. Die Medikamentenliste wurde in 13 % der Fälle als Informationslücke erkannt [10].

Zudem zeichnet sich aus den Ergebnissen der in diesem Artikel durchgeführten Studie eine hohe Relevanz von Vorbefunden und Medikamentenlisten, besonders für die stationären Patienten, ab, da bei über einem Drittel der Patienten ein Arztbrief oder eine Medikamentenliste im stationären Verlauf ergänzt wurde. Hier ließe sich mithilfe einer gut geführten Telematikstruktur und der ePA bzw. NFD eine deutliche Arbeitserleichterung und eine verbesserte Patientenversorgung erzielen.

Bei fast einem Viertel der im Verlauf stationär behandelten Patienten, bei denen es im Verlauf zu einer Ergänzung im Bereich des Arztbriefs kam, führten diese Informationen zu einer Veränderung von Diagnostik- oder Therapieentscheidungen. Bei ergänzenden Informationen zur Medikamentenliste waren dies sogar bei über einem Drittel (36 %) der Fall. Dies belegt die Relevanz solcher Informationen für eine zügige und optimale Behandlung von Notfallpatienten.

Gerade bei älteren Patienten mit langer medizinischer Vorgeschichte kommt es häufig zu einer längerdauernden Anamnese, wenn zur Vervollständigung Hausärzte oder Angehörige miteinbezogen werden müssen [6]. Durch das Einführen einer ePA/NFD ließe sich die Anamnesezeit und somit die Zeit bis zu einer adäquaten Behandlung verkürzen. Zusätzlich könnte eine Über-/bzw. Doppeldiagnostik vermieden werden.

Auch für den Bereich der Einflussnahme liefert die Studie von Stiell et al. Ergebnisse hinsichtlich der Tragweite. So wurde der Einfluss auf Diagnostikentscheidungen mit 74 % der 404 Patienten mit Informationslücken angegeben, bei den Therapieentscheidungen mit 32 % und bei den Verordnungsentscheidungen mit 39 %. In 48 % der Fälle, wo es zu einer Einflussnahme kam, wurde die Wichtigkeit der Information mit „*very important or essential*“ in 32 % mit „*somewhat important*“ und in 20 % mit „*not essential but potentially helpful*“ angegeben [10].

Zwei Studien, die den Einfluss von Electronic Health Records (EHR) auf chronische Erkrankungen untersuchten, kamen zum Ergebnis, dass es zu einer deutlichen Reduktion an Diagnostik und medikamentöser Anordnung kommt. Zugleich wurde eine Mortalitätsreduktion bei Vorhandensein von EHR nachgewiesen [3, 9]. Gerade bei den chronischen Erkrankungen sehen die Wissenschaftler einen eventuell wertvollen Zusammenhang zwischen einer verbesserten Behandlung von Patienten in Notaufnahmen, falls diese Krankheitsinformationen in elektronischer Form mitbringen.

## Limitationen

Um die ermittelten Untersuchungsergebnisse zu untermauern, wären folgende Änderungen im Studiendesign nötig:

- Durchführung einer randomisierten, kontrollierten Studie;
- unterschiedliche Notaufnahmen und deren Struktur zur Datenerhebung verwenden, um eine bessere Übertragbarkeit zu erzielen;
- ermitteln der jeweiligen Fachdisziplin und des Alters der in die Studie eingeschlossenen Patienten, um ggf. Unterschiede aufzuzeigen;
- ganztägliche Rekrutierungszeit zur Vermeidung von Selektionsbias.

## Ausblick

Hinsichtlich der Untersuchungsergebnisse kann der Notfalldatensatz und die elektronische Patientenakte einen sinnvollen Beitrag für die innerklinische Notfallversorgung liefern. Ressourcen könnten effektiver genutzt werden. Weitere Studien könnten hinsichtlich der Liegedauer der stationären Patienten interessante Daten liefern, in wie weit die elektronische Patientenakte Einfluss auf diese nimmt. Um den Einfluss und Nutzen der ePA/NFD auf die klinische Notfallversorgung belegen zu können, sollte im Rahmen der Einführung dieser Instrumente in Pilotregionen ein Prä-post-Vergleich an großen notfallmedizinischen Einrichtungen durchgeführt werden. Ein solcher Vergleich könnte auch mögliche Optimierungspotenziale dieser Instrumente unmittelbar aufzeigen und für den weiteren Ausbau sehr wertvoll sein.

## Fazit für die Praxis

Eine vorhandene medizinische Vorgeschichte in Form einer ePA oder eines NFD kann insbesondere bei Patienten in der Notaufnahme dazu dienen, die Anamnesezeiten zu reduzieren und eine zielgerichtete Therapie und Diagnostik dem Patienten zukommen zu lassen, um so die Patientensicherheit zu erhöhen. Der Ausbau einer ePA bzw. eines NFD sollte aus diesem Grund vorangetrieben werden, um somit zukünftig ein geeignetes Mittel bei einer effizienten und strukturierten Patientenversorgung in den heute schon überfüllten Notaufnahmen zu sein.
