# Erratum zu: Informationen zur medizinischen Vorgeschichte in der Notaufnahme

**DOI:** 10.1007/s00063-021-00817-0

**Published:** 2021-04-26

**Authors:** M. Lorsbach, A. Gillessen, K. Revering, C. Juhra

**Affiliations:** 1 Stabsstelle für Telemedizin, Universitätsklinikum Münster, Hüfferstr. 73–79, 48149 Münster, Deutschland; 2 Klinik für Innere Medizin, Herz-Jesu-Krankenhaus Hiltrup GmbH, Münster-Hiltrup, Deutschland; 3 Zentrale Notaufnahme, Herz-Jesu-Krankenhaus Hiltrup GmbH, Münster-Hiltrup, Deutschland

**Erratum zu:**

**Med Klin Intensivmed Notfmed 2020**

10.1007/s00063-020-00661-8

Der Beitrag „Informationen zur medizinischen Vorgeschichte in der Notaufnahme“ von M. Lorsbach, A. Gillessen, K. Revering und C. Juhra wurde ursprünglich am 10. Februar 2020 ohne „Open Access“ online auf der Internetplattform des Verlags publiziert. Die Autoren haben sich jedoch nachträglich für eine „Open-Access“-Veröffentlichung entschieden. Das Urheberrecht des Artikels wurde deshalb am 17. März 2021 in © The Author(s) 2020 geändert.

